# Endothelial ActRIIA inhibition protects the cardiac microvasculature in severe viral respiratory infection

**DOI:** 10.21203/rs.3.rs-6306417/v1

**Published:** 2025-04-01

**Authors:** Peng Xia, Sujin Lee, Kangsan Roh, Jason Griffith, Yirong Zhou, Edward Guzman, Yanxi Shi, Zihui Yang, Claire Castro, Haobo Li, Yugene Y. Guo, Abhilasha Singh, Rachel S. Knipe, Idris Raji, Jia Han Xu, R. Keith Babbs, ffolliott Fisher, Jennifer Lachey, Jasbir Seehra, Paul B. Yu, Se-Jin Lee, Daniel G. Anderson, Aaron Aguirre, Anthony Rosenzweig, Rajeev Malhotra, Jason D. Roh

## Abstract

Cardiac complications, including myocardial injury and dysfunction, are common in severe viral respiratory infections (VRI) and are associated with increased mortality ^1–3^ . The pathophysiology of VRI-induced myocardial injury is multifactorial, but frequently involves structural damage to the heart’s microvascular network that leads to subsequent myocardial ischemia and dysfunction ^4–6^ . Currently, there are no targeted therapies available to prevent or attenuate VRI-associated myocardial injury. Moreover, the molecular mechanisms driving the cardiac microvascular pathology in severe VRI are largely unclear. In this study, we identify increased endothelial cell (EC) activin type IIA receptor (ActRIIA) signaling as a key mediator of cardiac microvascular injury and pathologic remodeling in severe VRI. We show that genetic deletion of EC ActRIIA is sufficient to mitigate EC death and myocardial capillary loss in a murine model of severe influenza infection, which results in improved myocardial perfusion, cardiac function, and survival. We then provide proof-of-concept evidence for two novel pharmacological approaches to target EC ActRIIA pathophysiology in the treatment of VRI-induced cardiac dysfunction.

**Additional Declarations: Yes** there is potential Competing Interest. Jason Roh and Anthony Rosenzweig have a patent on ActRII inhibition in heart failure. Jason Roh receives research support from Keros Therapeutics. Anthony Rosenzweig is a scientific co-founder of Thyrv (unrelated to this work). Sujin Lee is a consultant for Biohaven (unrelated to this work). Paul Yu is a co-founder, consultant, and equity holder for Keros Therapeutics. R Keith Babbs, ffolliot Fisher, Jennifer Lachey, and Jasbir Seehra are employed by Keros Therapeutics and are shareholders of the company.

## MAIN

The COVID-19 pandemic of 2020–2023 highlighted the clinical relevance of cardiac complications in severe viral respiratory infection (VRI). Myocardial injury was reported in over 1/3 of hospitalized patients infected with SARS-CoV2[1], which was associated with up to 3-fold higher in-hospital mortality[1] that was further increased with concomitant cardiac dysfunction or heart failure (HF)[7–9]. Although this phenomenon was initially thought to be specific to SARS-CoV2, comparable rates of myocardial injury and HF have been reported with non-COVID VRI, including respiratory syncytial virus and influenza[2,3,10–14]. These findings, along with similar associations between disease severity and myocardial injury observed across multiple respiratory viruses[3,9,10,13,14], has led to an overarching hypothesis that shared indirect mechanisms, nonspecific to the virus itself, underlie part of the core pathophysiology of acute myocardial injury and dysfunction in severe VRI.

The etiology of myocardial injury in severe VRI is multifactorial and complex. While direct cardiac infection is rare, a rapidly growing body of evidence suggests that structural and functional changes in the cardiac microvasculature, including inflammation, thrombosis, leakage, and rarefaction, serve a central role in the causal pathophysiology of cardiac injury and dysfunction in VRI[5,15–21]. It is well established that heightened systemic inflammation caused by severe infections can induce endothelial cell (EC) dysfunction and apoptosis, leading to marked decrements in microvascular integrity and density in tissues throughout the body[6,14,18,22–28]. However, the molecular mechanisms driving this process in the heart in severe VRI are not well understood. Moreover, whether interventions that directly target this pathophysiology in VRI could improve outcomes is unknown.

Here, using a murine model of sublethal influenza infection, we show that severe VRI induces profound capillary loss and adverse microvascular remodeling in the heart that is associated with cardiomyocyte injury, systolic dysfunction, and upregulated EC transforming growth factor beta (TGFβ) family signaling. Using a combination of *in vivo* and *in vitro* influenza model systems, we identify increased activin signaling via the EC activin type IIA receptor (ActRIIA) as a key link between severe VRI and cardiac microvascular injury. Genetic and pharmacological inhibition of EC ActRIIA provide proof-of-concept evidence that targeting this pathobiology can attenuate myocardial injury and dysfunction in severe VRI.

## RESULTS

## Severe influenza infection causes diffuse capillary loss and adverse remodeling in the heart

The sublethal H1N1 influenza murine model is an established preclinical model of severe VRI that is associated with acute cardiac dysfunction[29]. However, whether it can be used to study the microvascular pathophysiology of VRI-induced myocardial injury is unclear. To first confirm a cardiac dysfunction phenotype in this model, we performed echocardiography in mice five days after infection. Influenza infection resulted in a modest trend toward decreased left ventricular ejection fraction (LVEF, 80 ± 2% vs 73 ± 4%, $p = 0.1$, Table S1), but a significant 38% decline in LV global longitudinal strain (GLS), a more sensitive metric of LV systolic function (Fig. 1A). Histopathologic assessment of the LV demonstrated evidence of cardiomyocyte injury and degeneration, increased myocardial fibrosis, and a marked 37% reduction in capillary density in influenza-infected mice (Fig. 1B). This was associated with a 28% decrease in EC numbers in the heart (Fig S1) and 2.5-fold decrease in myocardial perfusion (Fig. 1C).

To better characterize the morphologic changes occurring in the cardiac microvasculature with influenza infection, tissue clearing of the LV with 3D reconstruction of the microvascular network was performed. This not only confirmed a reduction in capillaries in influenza-infected animals, but also revealed compensatory dilatation of the remaining capillaries with a 41% increase in their luminal diameter (Fig. 1D). Notably, capillary VE-cadherin expression, which is a critical regulator of EC intercellular junction integrity[30], was reduced by 54% in influenza-infected mice (Fig. 1D), and inversely associated with a 15-fold increase in vascular permeability (Fig. 1E).

Cardiac and endothelial cell transcriptional profiling identify increased activin-A signaling as a key mediator of microvascular pathobiology in severe VRI

To gain insights into the mechanisms underlying cardiac capillary loss and increased vascular permeability in influenza-infected animals, we first performed transcriptional profiling in LV samples from these mice and vehicle-treated controls. RNA-Seq identified 1,507 upregulated and 2,371 downregulated differentially expressed genes (Table S2). Differential expression patterns of hallmark HF genes, including *Nppa* ($\log_2(FC) = 1.5$, $p_{adj}=3.8\times10^{-4}$), *Nppb* ($\log_2(FC) = 1.5$, $p_{adj}=9\times10^{-7}$), and *Myh7* ($\log_2(FC) = 3.9$, $p_{adj}=2\times10^{-37}$), corroborated the acute systolic dysfunction observed in influenza-infected mice (Table S2). Pathway analysis identified enrichment of multiple angiogenesis- and apoptosis-related processes in the influenza-infected mice (Fig. 1F, Table S3). Notably, TGFβ family-related pathways emerged as some of the most significantly dysregulated signaling pathways in influenza-infected mice ("cellular response to TGFβ stimulus", rich factor = 0.44, $p_{adj}$= 1.05 x$10^{-20}$; "response to TGFβ", rich factor = 0.43, $p_{adj}$= 1.25 x$10^{-20}$; Fig. 1F, Table S3).

Multiple ligands of the TGFβ superfamily have been implicated as important regulators of EC health and angiogenesis[31]. To identify which of these might be contributing to the cardiac microvascular phenotype observed in influenza-infected mice, we performed RNA-Seq on cardiac microvascular ECs (CMVEC) exposed to either plasma from influenza-infected mice or control uninfected mice. This not only provided a robust *in vitro* model system to study the effects of VRI-induced systemic inflammation on CMVEC, but also identified 1,795 upregulated and 2,119 downregulated differentially expressed genes in response to influenza infection (Fig. 2A, Table S4). Among the 33 TGFβ superfamily ligands, growth differentiation factor 15 (*Gdf15*) and activin A (*Inhba)* were the two most highly upregulated in CMVEC exposed to plasma from influenza-infected mice (Fig. 2A, Table S5). GDF15 has previously been shown to be cardioprotective in the context of VRI[32] and is generally considered pro-angiogenic[33,34]. However, the role of activin A in VRI is unclear[35–38], which prompted us to further investigate this candidate ligand.

To first determine if activin A signaling is indeed increased in CMVEC in influenza-infected mice, we isolated ECs from the hearts of these animals. Cardiac EC *Inhba* expression was 2.1-fold higher in influenza-infected mice (Fig. 2B). This was associated with several indicators of increased activin A signaling, including a 1.7-fold increase in *Fstl3* mRNA expression and 3.1- and 1.5-fold increases in SMAD2 and SMAD3 phosphorylation (Fig. 2B-C). Influenza-infected mice also exhibited a 1.7-fold increase in circulating activin A levels (Fig S2). Moreover, we found that increasing circulating activin A alone was sufficient to induce downstream activin A signaling, microvascular rarefaction, and moderate dysfunction in the hearts of otherwise healthy wild-type mice (Fig. 2D-G, Table S1). Collectively, these data strongly suggest that both autocrine and paracrine EC activin A signaling contribute to the cardiac microvascular and EC loss in severe influenza infection.

### Targeted inhibition of activin type IIA receptor improves cardiac microvascular endothelial cell survival and permeability

Activin A has been shown to impair angiogenesis in ischemic models[39,40] and induce apoptosis in non-EC populations via both AKT-dependent and -independent mechanisms[41–43]. However, its effects on the cardiac microvasculature in severe VRI are unknown. Given the marked cardiac EC and capillary loss observed in influenza-infected mice (Fig. 1, Fig S1), we hypothesized that increased activin A signaling might negatively impact CMVEC survival in severe VRI. Exposing CMVEC to plasma from influenza-infected animals not only increased activin A signaling, but also increased EC death and permeability (Fig. 2H-J). These cells exhibited a 1.8-fold increase in Poly ADP ribose polymerase (PARP) cleavage, a marker of EC apoptosis[44], and a 15% reduction in AKT phosphorylation, a critical regulator of EC

survival[45](Fig. 2K). To determine if inhibiting activin A signaling could potentially improve CMVEC survival in the context of severe VRI, we performed siRNA-mediated knockdown of *Acvr2a*, the gene encoding the major activin A binding receptor, activin type IIA receptor (ActRIIA) (Fig. 2L). In isolated CMVEC exposed to plasma from influenza-infected mice, *Acvr2a* knockdown resulted in a 23% increase in EC survival (Fig. 2M) and a 12% decrease in permeability (Fig. 2N), which was associated with a 55% decrease in cleaved PARP and 1.9-fold increase in AKT phosphorylation (Fig. 2O). Taken together, these results strongly suggested that inhibiting EC activin A/ActRIIA signaling could potentially improve EC survival and microvascular pathology in severe VRI.

### Endothelial-specific Acvr2a genetic deletion attenuates influenza-induced cardiac microvascular pathology

To further investigate the potential functional role of EC activin-A/ActRIIA signaling in influenza-induced cardiac microvascular pathology, we next generated EC-specific *Acvr2a* knockout mice (referred to as EC-*Acvr2a* KO) to test the hypothesis that EC ActRIIA inhibition might be cardioprotective in severe VRI. Cardiac EC *Acvr2a* mRNA expression was decreased by 97% in these mice, with no significant reduction in *Acvr2a* mRNA expression in non-EC cardiac cells (Fig. 3A). At baseline, no significant differences in cardiac function or microvascular morphology were observed in EC-*Acvr2a* KO mice, compared to littermate flox/flox controls (Fig. S3). While influenza infection induced similar reductions in cardiac capillary density, perfusion, and function in flox/flox mice, compared to infected wild-type mice, infected EC-*Acvr2a* KO mice remarkably exhibited almost complete protection from these pathologic processes (Fig. 3B-E). This was not only associated with preservation of healthy microvascular morphology in the heart (Fig. 3F), but also improved survival at one week post-infection (Fig. 3G). Notably, similar cardioprotective effects from severe influenza infection were not observed in cardiomyocyte-specific *Acvr2a* KO mice (Fig S4), suggesting that the cardiac benefits derived from ActRIIA inhibition in severe VRI are likely mediated more through the endothelium, as opposed to the myocytes.

### Therapeutic approaches to targeting EC ActRIIA signaling in severe VRI

To determine if EC ActRIIA signaling can be therapeutically targeted for VRI-related cardiac dysfunction, we tested two pharmacological approaches in the influenza model (Fig. 4A). The first approach utilized RKER-012 (Keros Therapeutics, Lexington, MA, USA), a murinized version of a novel, investigational modified ActRIIB-Fc ligand trap, which has high affinity for activin A and B[46]. In mice infected with influenza, RKER-012 decreased cardiac ActRII signaling, as indicated by a 30% decrease in *Fstl3* mRNA expression (Fig. 4B). This was associated with a decrease in mRNA expression of HF genes, *Nppa* and *Nppb*, along with preservation of LV GLS and overall improved survival at one week (Fig. 4C,D,G). Although activin inhibition by RKER-012 is not EC specific, it generated similar effects on the cardiac microvasculature compared to EC-Acvr2a KO mice, increasing myocardial capillary density by 36% and perfusion by 2.8-fold in influenza-infected animals (Fig. 4E-F).

To increase EC-specificity with targeted ActRII interventions, we next generated 7C1 nanoparticles encapsulating *Acvr2a* siRNA. 7C1 polymer-nucleic acid nanoparticles induce efficient EC-specific gene silencing *in vivo*[47]. In our murine model, we found that 7C1-*Acvr2a* nanoparticles decreased cardiac EC *Acvr2a* mRNA expression by 82%, but did not significantly reduce *Acvr2a* expression in non-EC cardiac cells (Fig. 4H). Similar to RKER-012, the 7C1-*Acvr2a* nanoparticles reduced cardiac *Fstl3, Nppa*, and *Nppb* mRNA expression and improved cardiac function in influenza-infected mice (Fig. 4I-K). Importantly, 7C1-*Acvr2a* nanoparticles protected mice from the acute myocardial capillary loss and impaired myocardial perfusion induced by severe influenza infection, improving overall survival (Fig. 4L-N).

## DISCUSSION

This study identified a novel role of increased EC activin A/ActRIIA signaling in the pathophysiology of cardiac microvascular injury and dysfunction in severe viral respiratory infection. Systemically activated inflammation/coagulation pathways represent the predominant link between primary lung infection and myocardial injury[4,10,18,21,48,49], which is largely mediated through EC injury and pathologic remodeling of the microvascular network in the heart[4–6,14,18–20,25,26]. While this microvascular hypothesis has gained popularity since the recent COVID-19 pandemic, the molecular mechanisms underlying it remain largely unclear. This study not only adds to our understanding of the molecular drivers of this pathologic process, but also provides proof-of-concept evidence that directly targeting mechanisms of myocardial injury could be an effective therapeutic approach to improving outcomes in severe VRI.

Here, using a series of gain- and loss-of-function approaches, in both *in vitro* and *in vivo* influenza models, we provide strong evidence supporting a functional role of increased EC ActRIIA signaling in the microvascular rarefaction and impaired myocardial perfusion/function that occurs in severe VRI. Importantly, we show that pharmacological reagents, either soluble ActRII-Fc ligand traps or nanoparticles encapsulated with *Acvr2a* siRNAs, can effectively inhibit cardiac ActRIIA signaling *in vivo* and provide robust cardioprotection that results in improved cardiac function and survival. To the best of our knowledge, these are the first data to show that targeted inhibition of EC ActRIIA signaling improves adverse cardiac microvascular remodeling and dysfunction. While this study focused on VRI, it is possible that the therapeutic benefits of EC ActRIIA inhibition could be relevant to other diseases affected by cardiac microvascular dysfunction, including syndrome X, heart failure with preserved ejection fraction, and stress- and pregnancy-related cardiomyopathies[50–53].

Our results also largely support a growing body of evidence implicating TGFβ family signaling as a critical regulator of microvascular health and function[31,38,54,55]. Activin A, in particular, has been shown to impair angiogenesis in skeletal muscle and lungs in the context of ischemia and hypoxia[39,40,56–58] and induce apoptosis in non-EC populations[41–43]. However, its role in the dynamic cardiac microvascular network has never been studied. In this study, we show that activin A has similar effects in the heart, and importantly, that the deleterious effects of systemic inflammation on CMVEC survival and function can be attenuated by targeted inhibition of activin A's primary receptor, ActRIIA. Notably, an ActRIIA-Fc ligand trap is approved by the FDA for pulmonary artery hypertension[59], highlighting the translational potential of the findings from this study. Moreover, 7C1 nanoparticles have been shown to be effective in EC gene silencing in nonhuman primates[47], suggesting that this novel EC-specific approach to ActRIIA inhibition could also be effective in human disease.

While our study provides new mechanistic insights into the pathobiology of cardiac microvascular and EC dysfunction in VRI with important therapeutic implications, several limitations are worth noting. First, we only tested the efficacy of EC ActRIIA inhibition in an influenza model. While we believe similar cardiac effects would be seen in other severe VRI, this would have to be directly tested in other VRI models to confirm. Second, the focus of our study was on cardiac microvascular pathology. The genetic and pharmacological ActRII interventions used in this study were systemic, and thus, they likely have effects on extra-cardiac tissues. Given the similar microvascular phenotypes reported in other tissues in VRI and related critical illness physiology[22,25,28,60,61], we would hypothesize that ActRII inhibition would have comparable effects in other microcirculatory networks. Further work exploring the systemic effects of EC ActRII inhibition will be important to assess potential therapeutic benefits in other organ systems that could also have crosstalk with the heart, as well as potential adverse effects of these interventions. Additionally, while our study evaluated the effects of EC ActRII signaling on clinically relevant structural and functional microvascular phenotypes in the heart, further studies exploring related pathobiology, such as vascular inflammation and thrombosis, will be important to fully evaluate the therapeutic potential of inhibiting ActRII signaling in VRI. Moreover, this study was done exclusively in preclinical models, and thus, safety and efficacy of this approach needs to be rigorously assessed in clinical studies. Lastly, we only studied the effects of ActRII inhibition in the acute infectious setting. Coronary microvascular function is impaired in both acute and chronic phases of COVID-19, and has been hypothesized as a potential contributor to the enigmatic pathophysiology of long COVID[4,21,25,26]. Whether the benefits of ActRII inhibition on the cardiac microvasculature could improve long term cardiopulmonary function after the index VRI are unclear and should be further investigated.

In conclusion, given the increased morbidity and mortality associated with myocardial injury and cardiac dysfunction in severe VRI, targeting causative mechanisms underlying the core microvascular pathophysiology of these cardiac complications could improve patient outcomes. Here we identify EC ActRIIA as a potential therapeutic target. Inhibition of EC ActRIIA signaling provides robust cardioprotection from acute VRI through preservation of the cardiac microvascular network. These findings not only provide a new therapeutic approach to addressing microvascular disease in severe VRI, but potentially also a broader spectrum of cardiac microvascular disease syndromes.

### METHODS

## Mice

All mice used in this study were on a C57BL/6J background. Endothelial-specific *Acvr2a* knockout (referred to as EC-*Acvr2a* KO for simplicity) were generated by crossing *Acvr2a* floxed mice[62] with *Tie2* Cre mice (Jackson Laboratory, #008863). Cardiomyocyte-specific *Acvr2a* knockout mice were generated by crossing *Acvr2a* floxed mice with *Myh6* Cre mice (Jackson Laboratory, #011038). All animal work was approved by the MGH IACUC.

### Murine interventions

To model severe influenza infection in mice, a sublethal dose of H1N1 influenza virus (7.5 x $10^7$/kg, Influenza A/PR/8/34, Charles River) was administered to 12–16-week-old mice via intranasal inoculation. Interventions targeting activin A/ActRIIA included: 1) Recombinant Activin A (0.1 mg/kg, intraperitoneally) or vehicle control (phosphate buffered saline, PBS) was administered daily for four consecutive days in the absence of H1N1 infection; 2) RKER-012 (10 mg/kg, subcutaneously) or an equivalent volume of vehicle control (tris buffered saline, TBS) was administered one day before H1N1 infection and again 24 hours prior to sacrifice; 3) An EC-specific nanoparticle-siRNA complex (0.8 mg/kg via tail vein injection) was administered one day before H1N1 infection.

## 7C1-Acvr2a siRNA Nanoparticle Construction

The 7C1-Acvr2a siRNA nanoparticles were constructed using previously published methods[63]. Briefly, siRNA-Acvr2a (Cat: # 4390818, ID: s61932; Negative Control: ID: 4390844, ThermoFisher) were incorporated into nanoparticles generated from PEI600 modified with C15 lipids. The nanoparticles were dialyzed against PBS and passed through a sterile filtration process to ensure purity and injectability prior to use *in vivo*.

## Murine echocardiography

Echocardiography was performed using a Vevo 3100 Imaging System (FUJIFILM Visualsonics). Mice were anesthetized with 1–2% isoflurane. Animals were imaged at a heart rate between ~ 350–500 bpm. LV dimensions, ejection fraction, and global longitudinal strain (GLS) were measured using Visualsonics proprietary software. Contrast echocardiography was done by intravenous administration of 50 µL of Vevo MicroMarker® Non-Targeted Contrast Agent (VS-11913) via the tail vein. Using ECG and respiration gating methods, single image segments were extracted from sequentially acquired images in correspondence with the cardiac time gating window during diastole, at which time cardiac-induced motion is minimal. Time to peak contrast uptake, starting from the first signal of contrast uptake in the LV anterior wall was measured using linear regression as perfusion time(seconds) to assess myocardial perfusion.

## Histology and immunohistochemistry

Standard Hematoxylin and Eosin (H&E) and Masson's Trichrome (fibrosis) staining was performed on mid-LV sections. Rabbit anti-mouse CD31 antibody (1:50; Cat: 77699, Cell Signaling Technology) was used to quantify capillary density. To assess vascular permeability, Evans blue dye (Cat: A16774.18, Thermo Scientific) was administered via tail vein injection 30 minutes before tissue collection. LV sections were then co-stained with anti-mouse CD31 antibody. ImageJ software (NIH) was used for image quantification. Three to six randomly selected sections per heart were used for the quantification.

## Whole-mount tissue staining

Anesthetized mice were perfused with ice-cold PBS prior to sacrifice and tissue collection. Hearts were fixed with 4% paraformaldehyde for two hours at 4°C and subsequently incubated in 10% donkey serum blocking solution. Primary antibodies used in the immunostaining included anti-CD31 (1:200, rabbit polyclonal, Cat: 557355, BD Pharmingen) and anti-VE-cadherin (1:200, goat polyclonal, Cat: AF1002, R&D system). Goat anti-Rabbit IgG Alexa Fluor 488 and Donkey anti-Goat IgG Alexa F647-conjugated secondary antibodies (Cat: 111-545-003 and 705-605-147, Jackson ImmunoResearch) were used, and nuclei were stained with DAPI (1:1000, Cat: D1306, Invitrogen). Cardiac tissue was cleared with tetrahydrofuran (Cat: 401757, Sigma-Aldrich), dichloromethane (Cat: L090000, Sigma-Aldrich), benzyl alcohol (Cat: 305197, Sigma-Aldrich) and benzyl benzoate (Cat: B6630, Sigma-Aldrich). Immunofluorescence images were acquired using a Leica TCS SP8 X Laser Confocal Microscope (Leica). LAS X software (Leica) was used for image acquisition and processing. High-resolution images of the stained whole-mount hearts were maximum intensity projections of tiled z-stack images taken at 5-μm interval throughout the entire thickness of the tissue. Vessel diameters were measured in at least three distinct segments, and the mean value was used to define the vascular diameter. To calculate the group value, the mean diameter of at least three vessels per heart was determined, with data pooled from all samples for statistical analysis. Vessel integrity was evaluated from VE-cadherin immunostaining. The VE-cadherin signal was quantified per unit area. For each mouse, three vessels were analyzed, and data from four mice per group were used for comparative analysis.

## Quantitative real-time polymerase chain reaction

Total RNA was extracted from tissue samples using TRIzol reagent, followed by reverse transcription to cDNA. We performed quantitative PCR using standard ΔΔCt method. The following primers were used: *Inhba* (F: GATGTGCGGATTGCTTGTGA; R: TCTTCTTGCCCAGGAGCACTA), *Acvr2a* (F: TTTCCGGAGATGGAAGTCAC; R: AGGAGGGTAGGCCATCTTGT), *Nppa* (F: GAGCAAATCCCGTATACAGTGC; R: ATCTTCTACCGGCATCTTCTCC), *Nppb* (F: GCTGCTGGAGCTGATAAGAGAA; R: GTTCTTTTGTAGGGCCTTGGTC), *Fstl3* (F: AACACTCGCCATGCGTTCT; R: CAGAATCCTCGGAGCCCATC), *Rps18* (F: CATGCAGAACCCACGACAGTA; R: CCTCACGCAGCTTGTTGTCTA).

## Immunoblotting

Protein was extracted using radioimmunoprecipitation assay buffer, following standard Western blotting procedures. The following antibodies were used: phosphorylated SMAD2/3 (pSMAD2/3) (Cat: 8828, Cell Signaling), total SMAD2/3 (Cat: 07-408, EMD), Poly (ADP-ribose) polymerase (PARP) (Cat: 9524, Cell Signaling), phosphorylated AKT (pAKT) (Cat: 13038, Cell Signaling), total AKT (Cat: 4691, Cell Signaling), and glyceraldehyde 3-phosphate dehydrogenase (GAPDH) (Cat: 2118, Cell Signaling) as a loading control. After incubation with appropriate secondary antibodies (Cat: 7074 (rabbit) and 7076 (mouse), Cell Signaling), protein bands were visualized using enhanced chemiluminescence and analyzed using ImageJ software for densitometric quantification.

## Endothelial cell assays

C57BL/6 Mouse Primary Cardiac Microvascular Endothelial Cells (CMVEC) were purchased from CellBiologics (Cat: C57-6024) and used between passages 3–5. CMVEC were cultured under conditions simulating influenza infection with 2.5% plasma from influenza-infected or PBS-injected mice ± *Acvr2a* siRNA knockdown. Knockdowns were done using siRNAs-Acvr2a (Cat: AM16708, ID 162112, ThermoFisher) or si-Negative Control (AM4611). EC phenotypes were assessed using the following assays. Cell Survival: EC viability was assessed using the CCK-8 assay (Cat: AB228554, Abcam), according to manufacturer's protocol. Permeability: Endothelial monolayer integrity was evaluated using a fluorescence-based permeability assay (Cat: ECM644, Sigma). ECs were grown to confluence on transwell inserts (0.4 μm pore size) and treated as per experimental conditions. FITC-dextran (40 kDa) was added to the upper chamber, and its passage to the lower chamber was measured over time.

## Flow cytometry

Mouse hearts were digested using a buffer containing Collagenase I, Collagenase XI, Hyaluronidase, DNase, HEPES buffer, $CaCl_2$, and PBS. After perfusion and extraction, hearts were minced and digested at 37°C for one hour. The resulting suspension was filtered through a 70 μm nylon filter, washed with FACS buffer, and centrifuged to collect cells. The cells were stained with FITC-CD45 (Cat: 30-F11, BioLegend) and PE-CD31 (Cat: 390, Thermo Fisher) antibodies on ice for 15 minutes. Following two washes, the labeled cells were analyzed using a BD FACSAria machine and FlowJo software.

## RNA sequencing

RNA sequencing was performed by Novogene. Data quality check was performed on the raw reads in FASTQ files. Read counts were then uploaded to R version 4.3.2 for downstream analyses. Gene counts fewer than 5 were filtered and the clean reads were mapped to the reference genome based on the Ensembl ID. Using the edgeR package, differential gene expression (DEG) analysis was performed to identify genes with a |log2 (fold change)| >0.5 and false-discovery rate (FDR)-corrected p-value < 0.05. Pathway analysis was performed by mapping the DEGs to the gene ontology (GO) database using the cluster Profiler R package, with GO terms with FDR-adjusted p-value of < 0.05 considered as significantly enriched.

## Statistical Analysis:

Statistical analyses were conducted using GraphPad Prism (version 9.0). Data are presented as mean ± standard error of the mean (SEM). Comparisons between two independent groups were analyzed using two-tailed Student's t-tests. For comparisons involving more than two groups, one-way ANOVA followed by Tukey's post-hoc test was employed. Two-way ANOVA was used to analyze the effects of two independent variables simultaneously. Survival data were analyzed using the Kaplan-Meier method, and differences between survival curves were assessed using the log-rank test. A p-value of less than 0.05 was considered statistically significant for all tests.

### Declarations

#### DATA AVAILABILITY:

The RNA-seq raw data will be deposited in the Gene Expression Omnibus. All data are available in the main manuscript and supplemental files.

#### CODE AVAILABILITY

All code used for the RNA-seq analysis will be publicly available.

#### ACKNOWLEDGEMENTS:

Funding for this work was provided by the NIH (T32HL007208 and K01AG080077 to P.X.; K76AG064328 and R01HL170058 to J.D.R.; R01HL142809, R01HL159514, and R01HL162928 to R.M.; R01HL135886 and R35HL155318 to A.R.; R01HL171201 to H.L; R01HL168138 to R.S.K.; R01HL144515 to A.D.A.; R61AI161805 to D.G.A.; P30CA14051 to D.G.A.; R01HL131910, R01AR057374 to P.B.Y.); AHA (24SCEFIA1253853 to H.L.; 22TPA969625 to R.M.; 24SFRNPCN1284382 and Merit Award to A.R.); Hassenfeld Scholars Award (to J.D.R.); Fred and Ines Yeatts Fund for Innovative Research (to J.D.R); Marble Center Funding (to D.G.A.); Harrington Discovery Scholar Award (to A.R.); Leducq Foundation Award (to R.M.); Wild Family Foundation (to R.M.).

#### COMPETING INTERESTS:

J.D.R. and A.R. have a patent on ActRII inhibition in heart failure. J.D.R. receives research support from Keros Therapeutics. A.R. is a scientific co-founder of Thyrv, unrelated to this work. S.L. is a consultant for BioHaven, unrelated to this work. P.B.Y. is a co-founder, consultant, and equity holder for Keros

Therapeutics, which develops therapies for cardiovascular, hematologic, and musculoskeletal diseases targeting bone morphogenetic protein and TGF-β signaling pathways. The interests of J.D.R. and P.B.Y. are reviewed and managed by Mass General Brigham in accordance with their conflict-of-interest policies. R.K.B., F.F., J.L., and J.S. are employed by Keros Therapeutics, and are shareholders of the company.

## Figures

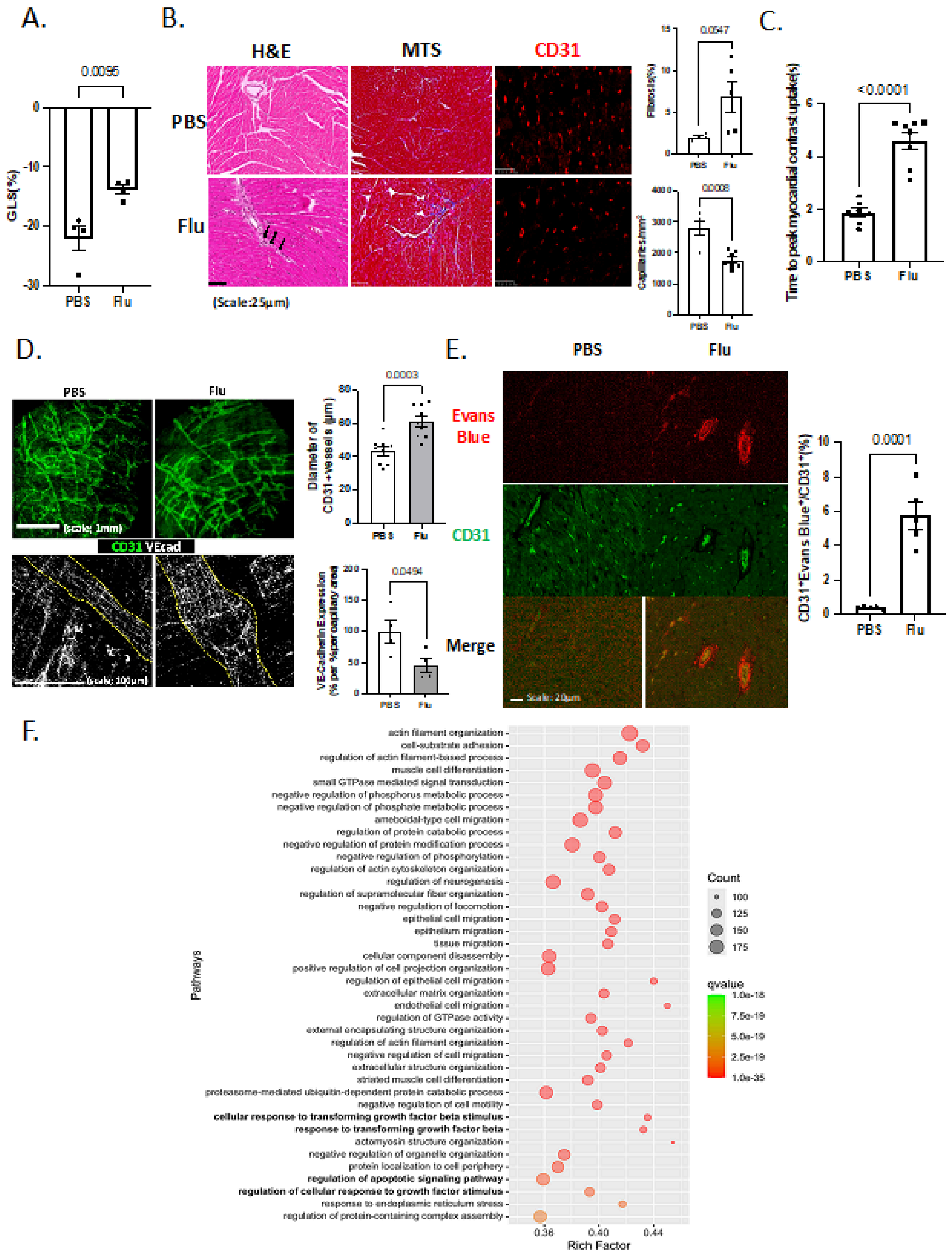

### Figure 1

**Severe influenza infection induces marked capillary loss and adverse microvascular remodeling in the heart**. Comparative analysis of mice infected with influenza (flu) versus vehicle control (PBS). All echocardiographic images were acquired at 5 days after infection. All molecular and histologic analyses done on tissue from day 6 after infection. (A) Global longitudinal strain (GLS), an echocardiographic metric of cardiac systolic function (more negative indicates better function). See Table S1 for complete echocardiography dataset. N=4/group. (B) Representative images and quantification of myocardial injury (H&E, arrow), fibrosis (blue= fibrosis) and capillary density (red= capillary). N=4-7/group. (C) Myocardial perfusion measured by contrast echocardiography (faster time to peak myocardial contrast uptake indicates better perfusion). N=7-8/group. (D) Representative images of 3D reconstruction of myocardial microvascular network done by tissue clearing with CD31 and VE-cadherin staining. Quantification of vessel diameter and VE-Cadherin expression. N=4-10/group. (E) Representative images and quantification of myocardial vascular permeability (CD31 (green)= vessel; Evans blue (red) = vascular leakage). N=5/group. (F) Pathway analysis of cardiac RNA-Seq from mice infected with flu vs PBS (n=3/group). Enrichment plot displays the 30 most significant dysregulated pathways. All bar graphs displayed as mean ± SEM with individual data points shown. Student's t-test used. $P < 0.05$ considered significant.

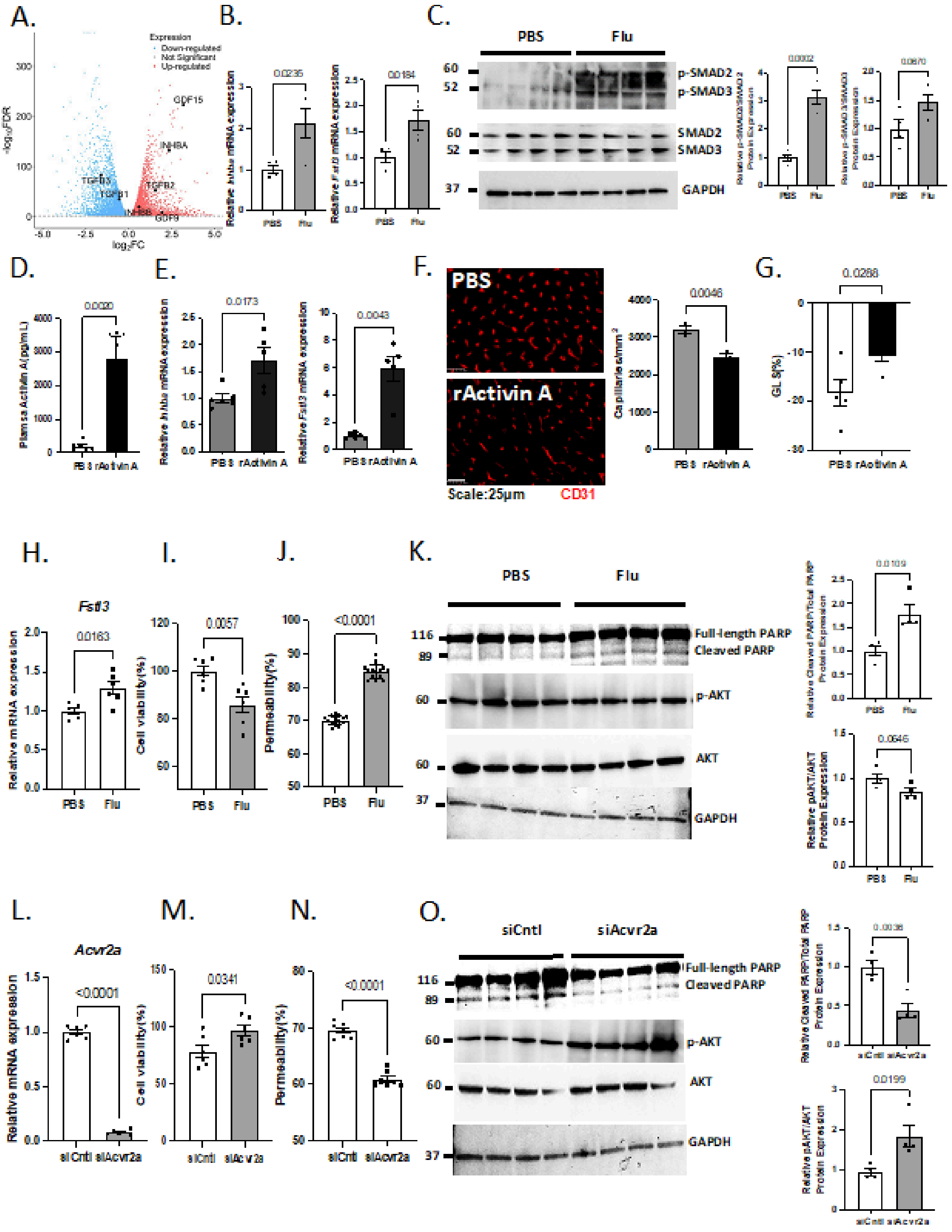

### Figure 2

**Activin-A/ActRIIA signaling regulates cardiac microvascular endothelial cell survival and vascular permeability in severe influenza**. (A) Volcano plot of differentially expressed genes in CMVEC exposed to 2.5% plasma from either flu-infected mice vs vehicle (PBS) controls. TGFb family ligands with a $log_2$(FC) >0.5 and FDR-adjusted p value <0.05 are highlighted in the plot. N=3/group. (B) Relative mRNA expression of *Inhba* (gene encoding activin A) and *Fstl3* (downstream activin signaling) in ECs isolated from hearts of mice infected with influenza vs PBS. Cardiac ECs collected three days post-infection. N=4/group. (C) Representative immunoblot and quantification of phosphorylated and total SMAD2/3 in isolated cardiac ECs. N=4/group. (D-G) C57BL/6J (14-week-old males) administered 0.1 mg/kg/day of recombinant activin A (rActivin-A) vs vehicle control (PBS) for four consecutive days. (D) Plasma level of activin A. N=5-6/group. (E) Relative mRNA expression of cardiac *Inhba* and *Fstl3*. N=5-6/group. (F) Representative images and quantification of myocardial capillary density (CD31= red). N=3/group. (G) LV GLS. See Table S1 for complete echocardiography data. N=5/group. (H-K) Mouse CMVEC cultured with 2.5% plasma from either flu-infected vs vehicle (PBS) control mice for 24 hours. (H) Relative *Fstl3* mRNA expression. N=6/group. (I) EC viability assay. N=6/group. (J) EC permeability assay. N=12/group. (K) Representative immunoblot and quantification of PARP Cleavage and phosphorylated AKT. N=4/group. (L-O) Mouse CMVEC exposed to 2.5% plasma from flu-infected mouse after siRNA knockdown of *Acvr2a* (si*Acvr2a*) vs siCntl. (L) Relative *Acvr2a* mRNA expression. N=6/group. (M) EC viability assay. N=6/group. (N) EC permeability assay. N=7/group. (O) Representative immunoblot and quantification of PARP Cleavage and phosphorylated AKT. N=4/group. All bar graphs displayed as mean ± SEM with individual data points shown. Student's t-test used. P <0.05 considered significant.

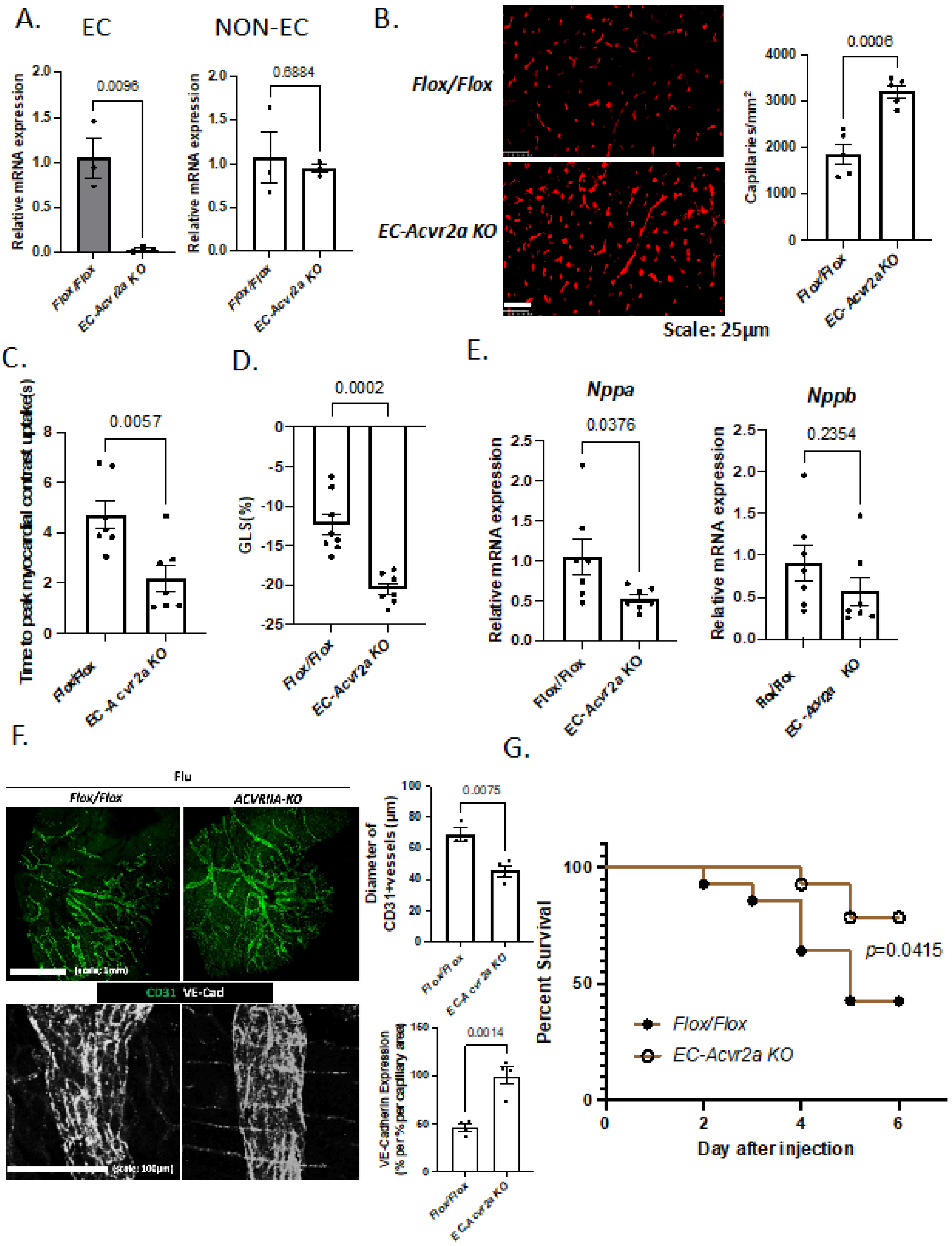

### Figure 3

**Endothelial-specific *Acvr2a* deletion attenuates influenza-induced cardiac microvascular pathology.** EC-*Acvr2a*-KO mice were generated by crossing *Acvr2a* flox/flox with *Tie2*-Cre mice. (A) Relative *Acvr2a* mRNA expression in cardiac endothelial cells (EC) and non-EC cells from EC *Acvr2a* KO mice vs flox/flox littermate controls. N=3/group. (B-G) Data from EC *Acvr2a* KO mice and flox/flox littermate controls infected with influenza. Echocardiography done at day 5 post-infection. Histologic and molecular analyses done on tissue collected at day 6 post-infection. (B) Representative image and quantification of myocardial capillary density (CD31= red). N=5/group. (C) Myocardial perfusion measured by contrast echocardiography. N=7/group. (D) LV GLS. See Table S1 for complete echocardiography data. N=7-8/group. (E) Relative cardiac mRNA expression of HF-related genes, *Nppa* and *Nppb*. N=7/group. (F) Representative images of 3D reconstruction of myocardial microvascular network done by tissue clearing with CD31 and VE-cadherin staining. Quantification of vessel diameter and VE-Cadherin expression. N=3-4/group. (G) Kaplan Meier survival curve with a log-rank comparison. Event = death or weight loss > 25% (based on IACUC protocol). N=14/group. All bar graphs displayed as mean ±SEM with individual data points shown. Student’s t-test used for A-F. $P < 0.05$ considered significant.

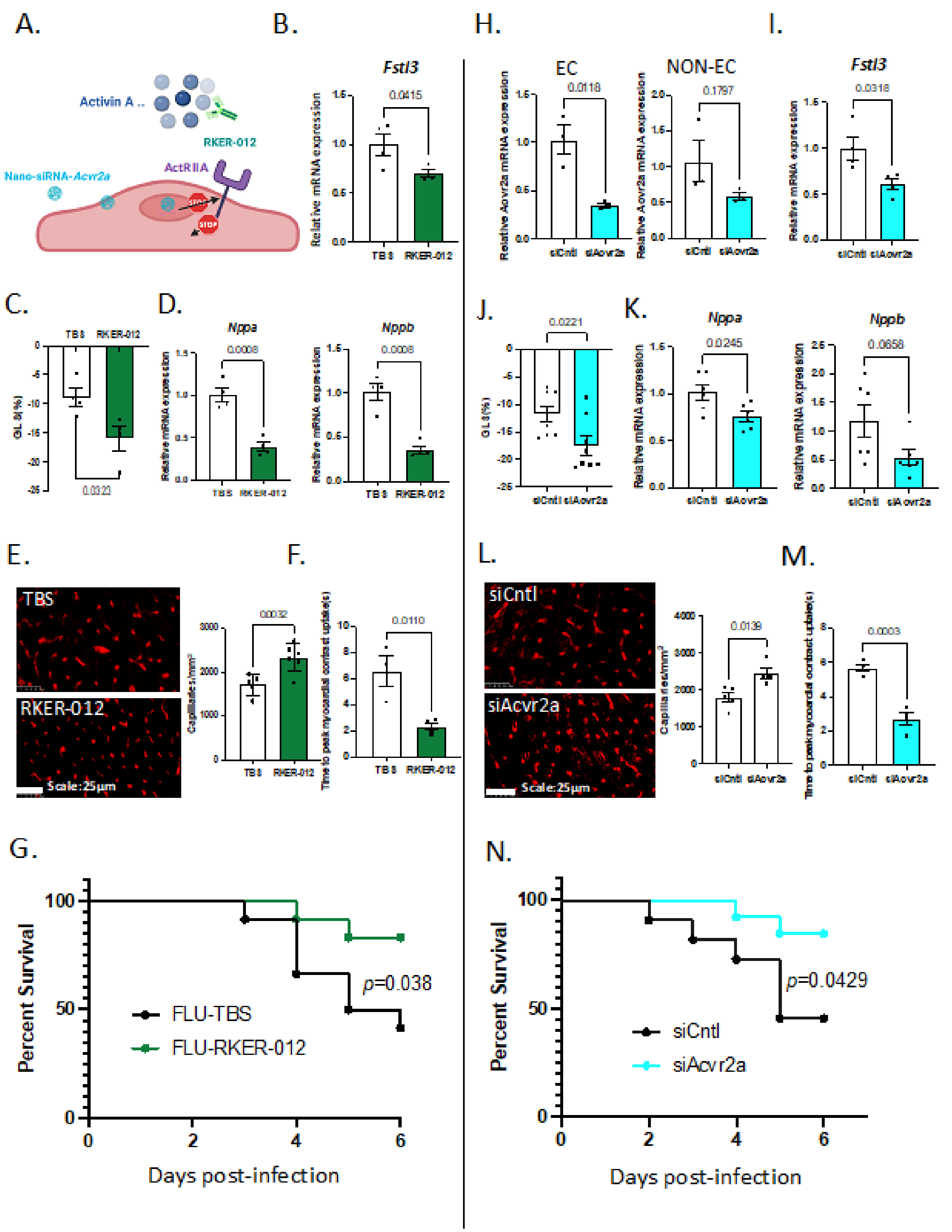

### Figure 4

**Pharmacological ActRII signaling inhibition improves cardiac microvascular integrity, myocardial perfusion and function, and overall survival in severe influenza**. (A) Schematic of pharmacological approaches to inhibiting ActRII signaling in severe VRI. RKER-012 (modified ActRIIB-Fc ligand trap). Nano-siRNA-*Acvr2a* (EC-specific nanoparticle delivery system of siAcvr2a); (B-G) RKER-012 intervention in mice infected with influenza. Echocardiography analysis done at day 5 and molecular/histology done at day 6 post-infection. (B) Relative cardiac *Fstl3* mRNA expression. N=4/group. (C) LV GLS. See Table S1 for complete echocardiography data. N=4/group (D) Relative cardiac mRNA expression of HF-related genes, *Nppa* and *Nppb*. N=4/group. (E) Representative image and quantification of myocardial capillary density (CD31= red). N=5-6/group. (F) Myocardial perfusion measured by contrast echocardiography. N=3-4/group. (G) Kaplan-Meier survival curve with a log-rank comparison. N=12/group. (H) Relative *Acvr2a* mRNA expression in isolated cardiac ECs and non-EC cells from non-infected mice. N=3/group. (I-M) Nano-siRNA-Acvr2a intervention in mice infected with influenza. (I) Relative cardiac *Fstl3* mRNA expression. N=4/group. (J) LV GLS. See Table S1 for complete echocardiography data. N=7-8/group. (K) Relative cardiac mRNA expression of HF-related genes, *Nppa* and *Nppb*. N=6/group. (L) Representative image and quantification of myocardial capillary density (CD31= red). N=4-5/group. (M) Myocardial perfusion measured by contrast echocardiography. N=4/group. (N) Kaplan-Meier survival curve with a log-rank comparison. N=11-13/group. Student's t-test used, except G and N. P <0.05 considered significant.

## Supplementary Files

This is a list of supplementary files associated with this preprint. Click to download.

- SupplementalTablesFINAL20250325.xlsx
- SupplementalFiguresFINAL20250325.docx
